# PDK1, associated with glycolytic metabolism, is a potential prognostic biomarker in osteosarcoma

**DOI:** 10.1371/journal.pone.0332494

**Published:** 2025-09-19

**Authors:** Jin Qi, Sihang Liu, Xufeng Hu, Xikang Luo, Yapeng Wang

**Affiliations:** 1 Department of Orthopedics, The First Affiliated Hospital of Wannan Medical College (Yijishan Hospital of Wannan Medical College), Wuhu, China; 2 Department of Orthopedics, The Second Hospital of Anhui Medical University, Hefei, China; 3 Department of Orthopedics, The Second Hospital & Clinical Medical School, Lanzhou University, Lanzhou, China; Southern Illinois University School of Medicine, UNITED STATES OF AMERICA

## Abstract

The study explores the prognostic significance and therapeutic potential of 3-phosphoinositide dependent protein kinase-1 (PDK1) in osteosarcoma. Using bioinformatics analysis and experimental validation, we analyzed PDK1 expression and its correlation with patient prognosis from GEPIA and UCSCXena databases. High PDK1 expression was found to be significantly associated with reduced survival in osteosarcoma patients, suggesting its value as a prognostic biomarker. Functional assays were performed to investigate the biological processes influenced by PDK1. Gene Ontology (GO) analysis indicated that PDK1 is involved in metabolism and cell proliferation. KEGG enrichment analysis revealed that genes related to PDK1 are enriched in pathways associated with metabolism, cell proliferation, and immune escape. In vitro experiments demonstrate that silencing PDK1 impairs glycolysis, reduces proliferation, and induces apoptosis in 143B osteosarcoma cells. Pan-cancer analysis confirms PDK1’s overexpression and poor prognosis association in multiple malignancies. Pan-cancer analysis extended the findings to other cancer types, confirming that PDK1 is overexpressed in multiple malignancies and generally associated with poor prognosis. This reinforces the potential of PDK1 as a universal biomarker and therapeutic target in oncology. The findings suggest PDK1 as a critical regulator of glycolytic metabolism and a potential therapeutic target in osteosarcoma. Targeting PDK1 could provide a novel therapeutic strategy for treating osteosarcoma and possibly other cancers.

## Introduction

Osteosarcoma is the most common primary malignant bone tumor, primarily affecting children and adolescents [1]. Despite advances in surgical techniques and adjuvant chemotherapy, the prognosis for osteosarcoma remains poor, especially for patients with metastatic disease [2]. The five-year survival rate for patients with localized osteosarcoma is approximately 60%, but it drops to 20–30% for those with metastatic disease [3]. Therefore, identifying reliable prognostic biomarkers and novel therapeutic targets is crucial for improving the clinical outcomes of osteosarcoma patients.

Metabolic reprogramming, a hallmark of cancer, plays a vital role in tumor progression and survival. Cancer cells often rely on glycolysis for energy production, even in the presence of oxygen, a phenomenon known as the Warburg effect [4,5]. One of the key regulators of this metabolic switch is the pyruvate dehydrogenase kinase (PDK) family, which inhibits the activity of the pyruvate dehydrogenase complex, thereby promoting glycolysis over oxidative phosphorylation [6]. Among the four PDK isoforms (PDK1–4), PDK1 has been shown to be overexpressed in several cancers, including ovarian cancer [7,8], gastric cancer [9], colorectal cancer [10], prostate cancer [11], and acute myeloid leukemia [12]. Its overexpression is generally associated with poor prognosis and increased tumor aggressiveness.

The role of PDK1 in cancer metabolism underscores its significance as both a prognostic biomarker and a therapeutic target. Understanding the specific mechanisms by which PDK1 regulates tumor metabolism and survival can pave the way for novel treatment strategies aimed at improving clinical outcomes for cancer patients. The specific role of PDK1 in osteosarcoma, particularly its impact on glycolytic metabolism and clinical outcomes, remains underexplored.

This study aims to investigate the expression and prognostic significance of PDK1 in osteosarcoma using bioinformatics analysis of publicly available datasets and experimental validation. We utilized the GEPIA and UCSCXena databases to analyze PDK1 expression in osteosarcoma and its correlation with patient prognosis. Additionally, we conducted in vitro experiments to examine the effects of PDK1 silencing on glycolytic metabolism in osteosarcoma cells.

Our findings reveal that high PDK1 expression is associated with significantly reduced survival in osteosarcoma patients, indicating its potential as a prognostic biomarker. Furthermore, silencing PDK1 in osteosarcoma cells impaired glycolysis, reduced cell proliferation, and induced apoptosis, highlighting its potential as a therapeutic target in osteosarcoma treatment. In addition, we compared the expression of PDK1 with that of classical osteosarcoma markers and found parallel expression patterns, suggesting that PDK1 may serve as a complementary or alternative prognostic biomarker for osteosarcoma.These results suggest that PDK1 plays a critical role in regulating the glycolytic metabolism of osteosarcoma cells and may serve as a potential therapeutic target.

By elucidating the role of PDK1 in osteosarcoma, this study provides valuable insights into the metabolic mechanisms underlying osteosarcoma progression and identifies PDK1 as a promising prognostic biomarker and therapeutic target. Future study should focus on further elucidating the molecular pathways regulated by PDK1 in osteosarcoma and exploring its potential in clinical applications to improve patient outcomes.

## Materials and methods

### Cell lines and culture conditions

Human osteosarcoma cells lines MG63 (cat.iCell-h140), U-2 OS (cat. iCell-h218), and 143B (cat. iCell-h231), andthe human osteoblast cell line hFOB1.19(cat. iCell-h094) were purchased from iCell Bioscience (Shanghai, China). All cell lines were cultured in Dulbecco’s Modified Eagle Medium (DMEM,cat. 10–013-CVRC; Corning) supplemented with 10% fetal bovine serum (FBS, cat.A6904-500 ml; Invigentech) and 1% penicillin-streptomycin dual antibody(cat. CCS30032.01; Jiangsu Enmoasai Biotechnology). Cells were maintained in a humidified incubator at 37°C with 5% CO_2_, as previously described [13].

### Data collection

Transcriptomic and clinical data of osteosarcoma were obtained from the UCSCXena database (http://xena.ucsc.edu/). After excluding samples with missing or zero survival time values, 88 osteosarcoma cases were included for analysis. Notably, the expression data represent tumor tissues collected at the time of surgery, and thus are not influenced by post-surgical survival outcomes. However, comparing gene expression between “deceased” and “alive” groups can help identify potential prognostic biomarkers. Detailed clinical information is provided in S1 Table.

### Survival analysis and model evaluation

Samples were stratified into high and low PDK1 expression groups based on the upper quartile (top 25%) of expression levels across all osteosarcoma samples(the cut-off value of PDK1 was 9.47)*.* Kaplan-Meier survival curves were generated using the R “survival” package, and model performance was assessed using time-dependent ROC curves. Patients were further stratified into metastasis, non-metastasis, “alive” and “deceased” subgroups for subgroup analysis. In our survival analysis, the terms “alive” and “deceased” refer to patient outcomes. Specifically, “alive” indicates patients who survived the follow-up period, while “deceased” refers to those who passed away during the follow-up. These terms are used to explore whether there are differences in PDK1 expression levels between patients who survived (“alive”) and those who died (“deceased”). The goal is to investigate potential associations between PDK1 gene expression and patient prognosis. Importantly, the gene expression data were generated from tumor samples collected during surgery, and the patient’s subsequent survival status does not influence the integrity or quality of the sequenced tumor samples.

### GO functional annotation and KEGG analysis

Gene enrichment analyses were performed using the LinkedOmics database (http://linkedomics.org/). The top 50 genes positively and negatively correlated with PDK1 were selected for GO enrichment analysis. Significance was defined as adjusted p-value (padj) < 0.05 and correlation coefficient > 0.5. KEGG pathway enrichment was conducted using DAVID v6.8 [14].

### Pathway enrichment analysis

To further explore the biological pathways associated with PDK1-related genes, we performed functional enrichment using Reactome pathway databases(https://reactome.org/). Significant pathways were identified using adjusted p-values < 0.05.

### Pan-cancer analysis

Normalized expression data from TCGA-GTEx (PANCAN, N = 19131, G = 60499) were downloaded from UCSC (https://xenabrowser.net/). PDK1 expression was compared between tumor and normal tissues across 32 cancer types using the Wilcoxon rank-sum and signed-rank tests.Disease-free interval data were included for prognostic evaluation.

### A novel nomogram constructed based on the risk signature

Based on the risk signature and clinicopathological features, a novel nomogram was constructed to predict the prognosis of osteosarcoma using variables with p < 0.05 in the multivariate Cox model. The predictive accuracy of the model was evaluated by generating a calibration curve [15].

### Cell transfectionLipofectamine

3000(cat. L3000008;Thermo Fisher) is used to transfect plasmids or siRNA-PDK1 into 143B cells [16]. Transfection was performed when cells reached ~60% confluence. Reagents were mixed in serum-free DMEM as per the manufacturer’s protocol and incubated at room temperature. After a 4-hour transfection period, the medium was replaced with complete culture medium.

### Cell Proliferation Assay (CCK-8)

143 B cells (1000 cells/well) were seeded in 96-well plates.At 24, 48, and 72 hours, 10 μL of CCK-8 solutions (cat. IV08–100; Invigentech) was added to each well and incubated for 2 hours at 37˚C. finally the absorbance value was measured at 450 nm using a microplate reader [17].

### Flow cytometry for apoptosis and cell cycle analysis

143B cells were harvested using 0.25% trypsin (cat. 9002-07-7; Merck), washed with PBS (cat. WH0112201−911XP, Procell), and resuspended. Apoptosis and cell cycle were analyzed using commercial kits (Apoptosis: cat. FMSAV647−100; Fcmacs; Cell cycle: cat. C1052; Beyotime) according to the manufacturers’ protocols. Samples were analyzed using FlowJo v10.0.7 [18].

### Cloning formation assay

143B cells (2,000 cells/well) were seeded into 6-well plates and cultured for 5–7 days in complete medium. Colonies were fixed with 4% paraformaldehyde (cat. S19004; Yuanye), stained with crystal violet, and imaged.

Glycolysis-Related Metabolic AssaysThe experimental methods related to energy metabolism refer to previous study [19]. Transfected and control 143B cells (4 × 10⁵ cells/well) were seeded in 6-well plates and cultured for 24 hours. Glucose, lactate, ATP, and glucose-6-phosphate (G6P) levels were measured using commercial kits: glucose (cat. S0201S; Beyotime), lactate (cat. G0816W; Grace), ATP (cat. E-BC-F002; Elabscience), and G6P (cat. S0185 Beyotime).

### Reverse transcription-polymerase chain reaction (RT-PCR)

RT-PCR analysis was carried out as described previously [20]. Total RNA was extracted from 5 × 10^5^ 143B cells using Trizol reagent (cat. 15596018; Life Technologies), and cDNA was synthesized using the first stranded cDNA synthesis kit (cat.mf166 plus 01; Mei5bio), RT-PCR was performed using M5 HiPer SYBR Premix Es Taq (Mei5bio, cat. MF787−01) with standard cycling conditions (94°C for 15 s, 60°C for 25 s, 72°C for 10 s; 40 cycles). Primers are listed in S2 Table.

### Western Blotting (WB)

When cell density reached ~90%, cells (1 × 10⁶) were lysed, WB analysis have been described in previous study [9]. In brief, When cell growth reaches a density of about 90% (1 × 10^6^) were lysed, and total proteins were extracted and quantified. Proteins were separated by SDS-PAGE and transferred to PVDF membranes (cat. ISED00010; Millipore). Membranes were blocked with 5% skim milk and incubated with PDK1 primary antibody (1:2000; Proteintech, cat. 18262–1-AP), followed by HRP-conjugated goat anti-rabbit secondary antibody (1:10,000). Detection was performed using ECL reagents, and signals were visualized using a chemiluminescence system.

### Statistical analysis

The software and plotting software used for data statistical analysis are Graphpad Prism 5 (Graphpad Software, San Diego, CA), with Flowjo and Modfit as the flow plotting software. Data are presented as mean ± standard deviation (S.D.). Differences between groups were analyzed using one-way ANOVA or two-way ANOVA for differential analysis and Dunnett test. The screening criteria for significant and extremely significant differences are* p < 0.05, **p < 0.01 and***p < 0.001.

## Results

### Osteosarcoma patients’ characteristics

From UCSCXena database, a total of 101 patients with osteosarcoma were included in this study, of which 25 patients had metastasis and 78 patients younger than 18 years old. The baseline data of the patients are shown in Table 1. Delete samples of osteosarcoma patients with missing or zero survival time values, and ultimately select 88 osteosarcoma patients as the research subjects for subsequent analysis. The detailed clinical information of 88 patients with osteosarcoma can be found in S1 Table, and the detailed expression matrix of 88 patients with osteosarcoma can be found in S3 Table.

**Table 1:** The characteristic of patients with osteosarcoma from UCSCXena database.

The Clinical data of patients with osteosarcoma		
Characteristic	levels	Overall
n		101
Metastasis, n (%)	No	76 (75.2%)
	Yes	25 (24.8%)
Age, n (%)	<18	78 (77.2%)
	>=18	23 (22.8%)
Gender, n (%)	Female	41 (40.6%)
	Male	60 (59.4%)
osteosarcoma event, n (%)	Alive	58 (58.6%)
	Deceaded	41 (41.4%)

**Fig 1:**
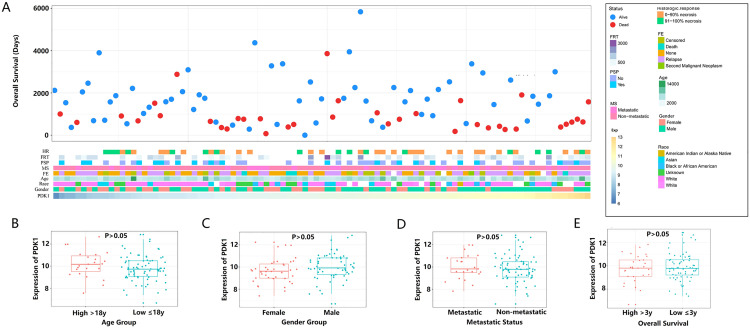
Association between PDK1 and clinicopathological characteristics of osteosarcoma. (A) The landscape of PDK1-related clinicopathological features in osteosarcoma from the UCSCXena database. No statistically significant differences in PDK1 expression were observed among the following subgroups: (B) Age group (>18 vs. ≤ 18 years), (C) Gender group,(D) Metastasis status group, and (E) 3-year survival status group. Statistical significance was assessed using an unpaired t test.

Patients with varying expression levels of PDK1 showed distinct patterns of clinical and pathological characteristics. Increases in PDK1, time to first relapse in days (FRT), primary site progression (PSP), metastasis status (MS), histologic response, first event (FE), age, gender and race showed asymmetric distributions in the UCSCXena datasets (Fig 1A). Comparative analysis was conducted with different groups of these samples. There was no statistically significant difference in PDK1 expression across the gender, age, metastatic status, and 3-year survival groups, although there was a trend of increased expression in the older (more than 18 years old) and metastatic groups(Fig 1B-1E).

### Correlation between PDK1 and patient survival status

**Fig 2:**
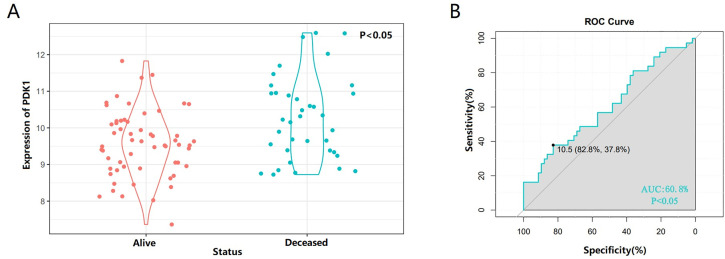
PDK1 is specifically enriched in the deceased group of osteosarcoma patients. (A) PDK1 was enriched in the deceased group of osteosarcoma patients in the UCSCXena databases. The significance of the difference was tested by unpaired t test. (B) The receiver-operating characteristic (ROC) curve showed the high-expression specificity of PDK1 in deceased group of osteosarcoma patients. AUC, area under the curve.

We further investigated whether PDK1 expression differed between patients who were alive or dead at last follow-up. We found that PDK1 was significantly enriched in the deceased groups (P < 0.05, Fig 2A), with data processing recorded in S4 Table. The receiver-operating characteristic (ROC) curve was performed to evaluate the expression specificity of PDK1 in the survival status of osteosarcoma patients. As expected, the area under the curve (AUC) was up to 60.8% in the UCSCXena database (P < 0.05, Fig 2B), These results suggested that PDK1 expression is closely related to the survival status of patients. Therefore, we need to explore why PDK1 abnormal expression phenomenon.

### Correlation analysis between PDK1 and prognosis of osteosarcoma

**Fig 3:**
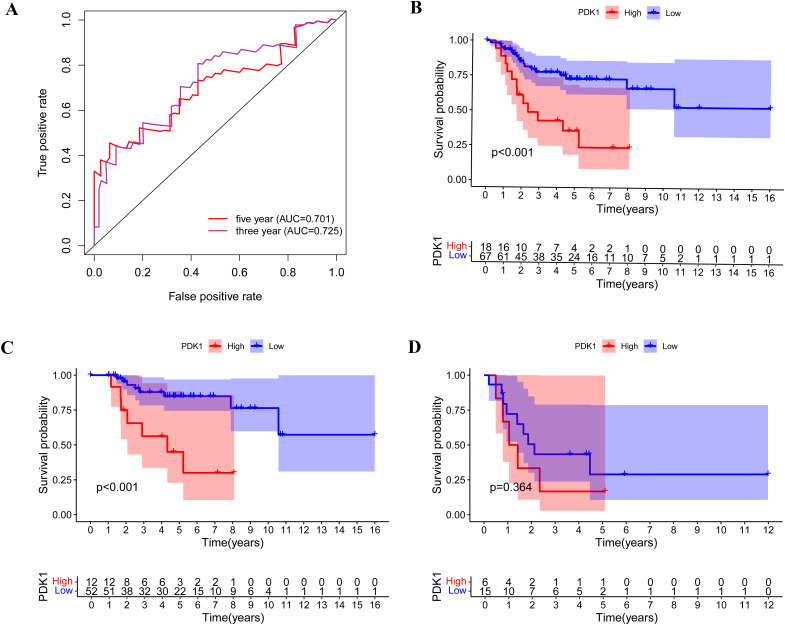
The correlation between the expression of PDK1 and prognosis outcomes in osteosarcoma. (A) AUC curve shows that the precision of this model is good. (B) Kaplan-Meier survival curves illustrate the increasing expression of PDK1 was negatively correlated with prognosis of patients with osteosarcoma. (C) The expression of PDK1 was negatively correlated with prognosis in non-metastic osteosarcoma group. (D)There was no significant correlation between PDK1 expression and prognosis in metastatic osteosarcoma group.

This study screened osteosarcoma samples with complete clinical follow-up data, established a model of PDK1 and clinical prognosis of osteosarcoma, and evaluated the accuracy of the model. PDK1 was divided into high expression group and low expression group (the cut-off value of PDK1 was 9.47). AUC curve showed that the model has good applicability in predicting the survival rate at three and five years (Fig 3A). Kaplan–Meier survival analysis showed that high expression of PDK1 was associated with poor prognosis of osteosarcoma.(P < 0.001, Fig 3B). We further divided the samples into non-metastasis group and metastasis group for subgroup analysis, in the non-metastatic group, patients with low PDK1 expression had longer overall survival (P < 0.001, Fig 3C). While in the metastasis group, the expression of PDK1 had no significant correlation with the prognosis (P > 0.05, Fig 3D). In conclusion, we speculate that the expression of PDK1 is negatively correlated with the overall sample of osteosarcoma and the prognosis of non-metastatic group.

### Role of PDK1 in osteosarcoma progression and metabolism

**Fig 4:**
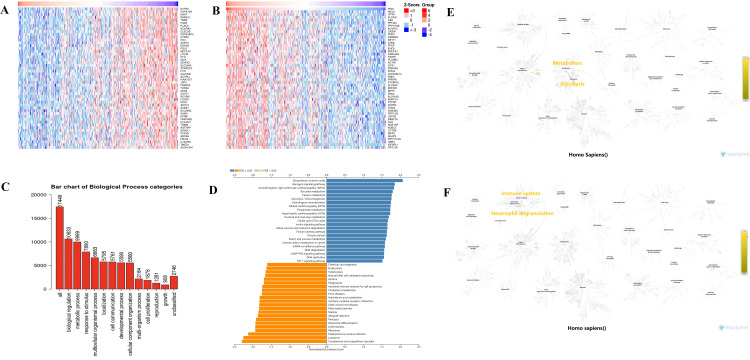
GO functional and KEGG pathway enrichment analysis of PDK1. (A) Top 50 genes most negatively associated with PDK1 are shown in a heatmap. (B) Top 50 genes most positively associated with PDK1 are shown in a heatmap. (C) GO functional annotation analysis showed that PDK1 played a key role in biological processes such as metabolism and cell proliferation. (D) KEGG enrichment analysis showed that positive related genes with PDK1 were mostly enriched in cell metabolism and proliferation pathways, while negative related genes were enriched in immune-related signaling pathways. (E) Reactome analysis of significant gene pathways negatively correlated with PDK1. (F) Reactome analysis of significant gene pathways positively correlated with PDK1.

To predict the function of PDK1, including associated pathways, we performed a positive and negative correlation analysis between PDK1 and other genes in osteosarcoma using LinkedOmic online database (Fig 4A-4B), with the detailed PDK1 related genes recorded in S5 Table. To further explore the role of PDK1 in osteosarcoma progression, we conducted GO enrichment analysis on PDK1, identifying biological processes such as metabolic processes and cell proliferation (Fig 4C). Our findings revealed that PDK1’s positively correlated genes are primarily involved in cellular metabolism and proliferation-related pathways, including amino acid biosynthesis, glycolysis and DNA replication. In contrast, negatively correlated genes are involved in immune-related pathways, such as endocytosis and NK cell-mediated cytotoxicity(Fig 4D). These findings suggest that PDK1 may promote tumor progression by enhancing cell metabolism and proliferation while potentially suppressing immune activity. To further validate the functional roles of PDK1-associated genes, we performed additional pathway enrichment analysis using the Reactome databases. Consistent with previous GO results, positively correlated genes were significantly enriched in metabolic and biosynthetic pathways, including “Glycolysis”, “Glucose metabolism” and “Gluconeogenesis”(Fig 4E, S6 Table). Meanwhile, negatively correlated genes were enriched in immune system pathways such as “Innate Immune System” and “Neutrophil degranulation” (Fig 4F, S7 Table). These findings further support the involvement of PDK1 in metabolic regulation and immune modulation in osteosarcoma.

### PDK1 expression correlates with key osteosarcoma biomarkers

**Fig 5:**
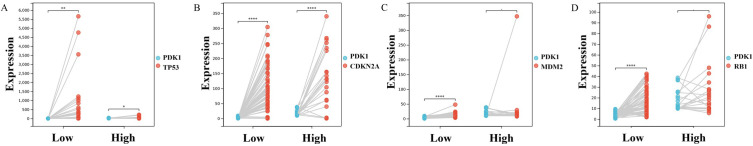
The expression of PDK1 shows a consistent trend with the expression levels of known osteosarcoma biomarkers. (A) Connected dot plot comparing TP53 expression with PDK1 expression between the PDK1-high and PDK1-low groups. (B) Connected dot plot comparing CDKN2A expression with PDK1 expression between the PDK1-high and PDK1-low groups. (C) Connected dot plot comparing MDM2 expression with PDK1 expression between the PDK1-high and PDK1-low groups. (D) Connected dot plot comparing RB1 expression with PDK1 expression between the PDK1-high and PDK1-low groups.

To further evaluate the relevance of PDK1 in osteosarcoma, we compared its expression with that of well-established osteosarcoma biomarkers, including TP53, CDKN2A,MDM2, and RB1, using the UCSCXena database. Stratification of samples by PDK1 expression revealed that TP53, CDKN2A,MDM2, and RB1 were significantly upregulated in the PDK1-high group compared to the PDK1-low group (p < 0.05, Fig 5A-5D). Although the absolute expression level of PDK1 was lower than these canonical markers, the consistent expression trends suggest co-regulation or shared upstream signaling pathways. Notably, CDKN2A,MDM2, and RB1 also appeared in the PDK1-related gene signature derived from our correlation analysis (S5 Table), further supporting the association between PDK1 and osteosarcoma oncogenic programs.

### The expression and prognostic significance of PDK1 in pan-cancer

**Fig 6:**
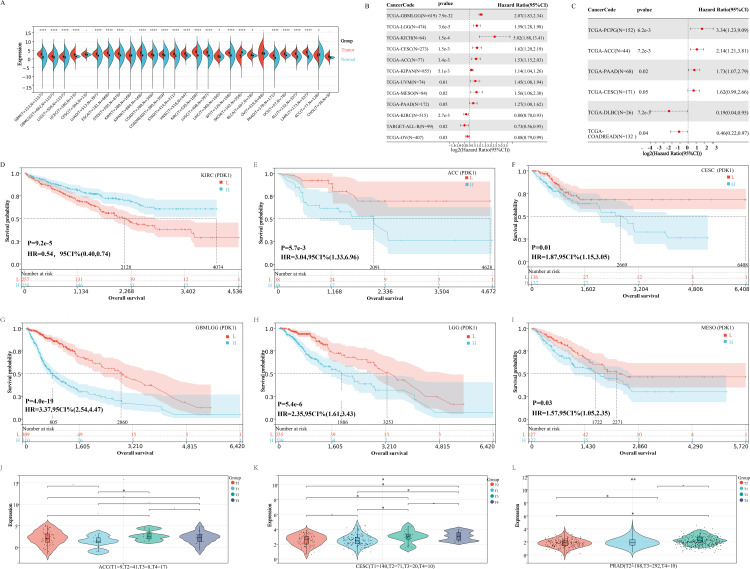
The expression and prognostic significance of PDK1 in different kinds of cancer. (A) Distributions of PDK1 expression levels are displayed using box plots. The statistical significance computed by the Wilcoxon test is annotated by the number of stars. (B) Overall survival analysis the prognostic relationship of PDK1 expression in different types of cancer by Cox proportional hazards regression model. (C) DFI analysis the prognostic relationship of PDK1 expression in different types of cancer by Cox proportional hazards regression model.(D-I) Pan-cancer Kaplan-Meier overall survival of PDK1 in indicated tumor types from TCGA database. The median value of PDK1 in each tumor was taken as the percentile (50%) of PDK1 expression.(J-L) Pan-cancer differential expression of PDK1 in WHO stages in indicatedumor types from TCGA database. *P < 0.05, **P < 0.01, ***P < 0.001.

To further determine whether the prognosis of PDK1 in pan-cancer is similar to that of PDK1 in osteosarcoma, we conducted pan-cancer analysis to evaluate the overall impact of PDK1 on patients with different types of tumors. We found that compared with normal tissues, PDK1 protein is highly expressed in BLCA, BRCA, CESC, ESCA, GBM, HNSC, KICH, KIRC, LIHC, LUAD, LUSC, PCPG, SKCM, THCA, and UCEC(P < 0.05, Fig 6A), with the detailed process recorded in S1 File. Moreover, PDK1 was a high-risk factor for patients with GBMLGG, LGG, KICH, CESC, ACC, KIPAN, UVM, MESO and PAAD, but a low-risk factor for patients with KIRC and ALL, as determined by overall survival analysis (Fig 6B). In addition, Disease Free Interval (DFI) analysis demonstrated that PDK1 was a high-risk factor for patients with PCPG, ACC, PAAD and CESC, but a low-risk factor for patients with DLBC and COADREAD (Fig 6C). We further divided patients into PDK1 high-expression group and PDK1 low-expression group based on the percentile (50%) of PDK1 expression, the results showed that only in KIRC patients, PDK1 was highly expressed and had a longer survival time, while in ACC,CESC,GBMLGG,LGG and SARC patients, PDK1 was highly expressed and had a shorter survival time (P < 0.05, Fig 6D-6I). Further analysis found the higher the tumor grade, the higher PDK1 expression in ACC/CESC/PRAD patients (P < 0.05, Fig 6J-6L), with the detailed analysis results recorded in S8 Table. These results indicate that PDK1 plays an important role as a poor prognostic factor in most types of cancer. Unfortunately, there is no transcriptome data for osteosarcoma in the UCSC database. To further study the expression of PDK1 in osteosarcoma and normal tissues, we immediately analyzed the expression of PDK1 in osteosarcoma cells and normal osteoblasts cells, as well as the function of PDK1 in osteosarcoma cells.

### Construction and calibration of an integrated monogram

**Fig 7:**
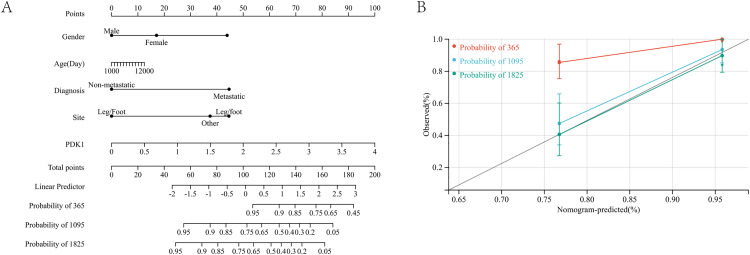
Construction and calibration of nomogram. (A) PDK1 expression and clinical features. (B)The calibration of the nomogram at 1, 3, and 5 years in the training cohort.

In this study, in order to more accurately predict the survival rates of osteosarcoma patients in one-year, three-years, and five-years, we integrated data on survival time, survival status, and five features including PDK1 expression to create a nomogram of patient survival rates (Fig 7A). Using the calibration curve to predict the effectiveness of the prediction model, the overall C-index of the model is 0.813, 95% CI (0.749–0.876), and P value = 2.37e-22 (Fig 7B).These results demonstrate that the integrated nomogram could predict the diagnosis of accuracy in osteosarcoma patients.

### PDK1 overexpression in osteosarcoma cells promotes glycolysis-dependent proliferation and invasion

**Fig 8:**
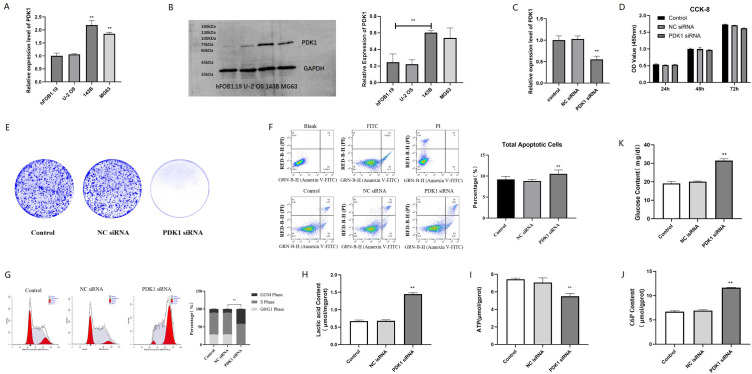
The expression and function of PDK1 in 143B cells. (A) Comparison of PDK1 mRNA expression between U-2 OS, 143B, MG63 and hFOB1.19 by qRT-PCR(n = 3). (B) Comparison of PDK1 protein expression between U-2 OS, 143B, MG63 and hFOB1.19 by western blotting(n = 3). (C) Analysis of PDK siRNA transfection efficiency in 143B cells by qRT-PCR(n = 3). (D) Detection of 143B viability at 24 hrs, 48 hrs and 72 hrs after silencing PDK1 by CCK-8 analysis(n = 3). (E) The effect of PDK1 siRNA on colony forming ability of 143B was detected by clone formation assay(n = 1). (F) Flow cytometry was used to detect the effect of PDK1 siRNA group and control group on apoptosis of 143B cells(n = 3). (G) Flow cytometry was used to detect the effect of PDK1 siRNA group and control group on cell cycle of 143B cells(n = 3). (H) Analysis of lactate content: The lactate produced during the glycolytic metabolism of 143B cells in the silenced PDK1 group and the control group(n = 3). (I) Analysis of ATP content: The ATP produced during the glycolytic metabolism of 143B cells in the silenced PDK1 group and the control group(n = 3). (J) Analysis of C6P content: The C6P produced during the glycolytic metabolism of 143B cells in the silenced PDK1 group and the control group(n = 3). (K) Analysis of glucose content: The glucose content during the glycolytic metabolism of 143B cells in the silenced PDK1 group and the control group(n = 3). *P < 0.05, **P < 0.01, ***P < 0.001.

Quantitative real-time polymerase chain reaction (qRT-PCR) and western blotting were used to analyse the transcription and translation levels of PDK1 in 143B cells. It was found that compared with osteoblast cell hFOB1.19, PDK1 expression was significantly increased in osteosarcoma 143B and MG63, while PDK1 only slightly increased in U-2 OS (Fig 8A, B), with original gel images showed in S2 File and data processing recorded in S9 Table. Subsequently, we chose 143B as the experimental osteosarcoma cells for the next validation experiment. qRT-PCR assay was used to detect the effect of PDK1 siRNA transfection in 143B. The results showed that after transfection with PDK1 siRNA, the expression level of PDK1 mRNA in 143B decreased (P < 0.05, Fig 8C). CCK8 analysis and cell cloning experiments were used to detect the effect of silencing PDK1 on the proliferation and invasion ability of 143B. CCK8 analysis showed that compared with the control group, silencing PDK1 did not significantly reduce the proliferation ability of 143B within 72 hours (P > 0.05, Fig 8D). The results of cell cloning experiments showed that after 2 weeks of cell culture, silencing PDK1 significantly weakened the vitality of 143B cells (Fig 8E).

Further use flow cytometry to detect the effects of silencing PDK1 on the cell cycle and apoptosis of 143B cells. The experiment found that silencing PDK1 caused 143B cells to stagnate in the G2/M phase and induced certain cell apoptosis, but the proportion of apoptosis was not very high, indicating that silencing PDK1 had a greater impact on 143B cell cycle arrest than cell apoptosis (Fig 8F, 8G).

GSEA found that PDK1 plays an important role in regulating the glucose metabolism of osteosarcoma cells. Therefore, the validation experiment analyzed the effect of silencing PDK1 on the metabolism of osteosarcoma cells. The experiment found that silencing PDK1 significantly reduced the ATP content in 143B cells and significantly increased the content of lactate, C6P and glucose (Fig 8H-8K), with the detailed process recorded in S10 Table, indicating that silencing PDK1 will hinder the glycolysis process of 143B. These results demonstrate that PDK1 drives glycolysis, thereby enhancing osteosarcoma cell proliferation and invasion.

## Discussion

The progression of osteosarcoma, like many cancers, cannot be separated from energy metabolism. The classic metabolic mode of energy metabolism in malignant tumors is glycolysis [21]. Even in non-hypoxic environments, cells still generate energy through a glycolysis pathway that is less efficient than the tricarboxylic acid cycle (TCA cycle) pathway. This high-speed but low efficiency ATP is an aspect of evolution that allows cancer cells to gain an advantage in competing for shared energy [22]. Meanwhile, high-speed glycolysis can also provide necessary intermediates for the synthesis and metabolism of cancer cells [23]. This kind of metabolic product of glycolysis is lactate, a phenomenon known as the Warburg effect [24]. PDK1 plays a major role in glycolysis and is closely related to tumor metabolism reprogramming. As an essential limiting enzyme for glycolysis, PDK1 can efficiently phosphorylate pyruvate dehydrogenase (PDH), promoting aerobic glycolysis in cancer cells and reducing cell damage caused by ROS accumulation [25]. In this study, we found that PDK1 expression did not significantly differ across gender, age, metastatic status, and 3-year survival groups, although there was a trend of increased expression in older and metastatic patients. We also found that PDK1 is involved in various metabolic processes in osteosarcoma cells, including glycogenesis, nitrate metabolism, dysregulated metabolism, tetraenoid backbone biosynthesis, alanine aspartate, and glutamate metabolism. Importantly,inhibiting PDK1 can interfere with the cell cycle of osteosarcoma, causing osteosarcoma cells to stagnate in the G2/M phase. Simultaneously inhibiting PDK1 can induce apoptosis of osteosarcoma cells. Survival analysis demonstrated a significant negative correlation between high PDK1 expression and overall survival in osteosarcoma patients. The ROC curve further validated the specificity of PDK1 expression in predicting survival status, with an AUC of 60.8%. Subgroup analysis revealed that low PDK1 expression was associated with longer survival in non-metastatic patients, indicating its prognostic value is particularly relevant in this subgroup.

Studies have shown that PDK1 can promote cell cycle progression by activating the PI3K/Akt, mTOR, and GSK-3β pathways to promote the expression of cyclin [26,27]. PDK1 synergistically promotes the expression of cyclin D1, which is a key factor driving cells from G1 phase to S phase [28]. Through this approach, PDK1 helps maintain normal cell proliferation and growth. For tumor cells, this means that inhibition of PDK1 can hinder the progression of the cell cycle, leading to slowed or stagnant tumor cell proliferation, thereby preventing its excessive proliferation and exhibiting potential anti-tumor effects. Meanwhile, PDK1 can inhibit cell apoptosis, and silencing PDK1 can reduce NF-kappaB pathway, thereby weakening anti-apoptotic signals and making tumor cells more susceptible to programmed cell death [29]. This includes promoting the activity of apoptosis promoting factors (Bax, Bak) and/or inhibiting the expression of apoptosis inhibiting factors (Bcl-2, Bcl-xL).

Interestingly, PDK1 expression also mirrored the expression of several classical osteosarcoma-associated markers, including TP53, CDKN2A, MDM2, and RB1. These genes have been extensively implicated in osteosarcoma tumorigenesis. TP53, one of the most frequently mutated genes in osteosarcoma, plays a central role in DNA damage response and apoptosis regulation, its inactivation promotes genomic instability and tumor progression [30]. CDKN2A, which encodes the tumor suppressors p16^INK4a and p14^ARF, is often deleted or epigenetically silenced in osteosarcoma, leading to cell cycle dysregulation [31]. MDM2, a negative regulator of TP53, is frequently amplified in osteosarcoma and contributes to tumor proliferation by promoting p53 degradation [32]. RB1, another key tumor suppressor, is involved in the control of G1/S cell cycle transition, and its loss is associated with poor prognosis and increased metastatic potential in osteosarcoma [33].Although the absolute expression level of PDK1 was lower than that of these canonical markers, the parallel expression patterns suggest that PDK1 may act in concert with these proteins, contributing to the regulation of cell proliferation and tumor progression. This finding further supports the potential clinical utility of PDK1 as a biomarker for osteosarcoma.Although our study focused on the effects of PDK1 silencing in osteosarcoma, multiple studies have shown that PDK1 overexpression enhances tumor aggressiveness. For example, PDK1 promotes EMT and metastasis in nasopharyngeal carcinoma, increases CD47 expression to facilitate immune escape in melanoma, and drives proliferation and invasion in ovarian and hypopharyngeal cancers [34–36]. These findings reinforce the oncogenic potential of PDK1 and support its role as a candidate therapeutic target and prognostic marker in diverse malignancies. PDK1 is a pleiotropic kinase involved in various physiological and oncogenic pathways, including the regulation of Akt pathway [37,38]. Its inhibition may result in both therapeutic benefits and off-target effects. PDK1 inhibitors, such as BX-795 and GSK2334470, have shown promise in preclinical studies across other tumor types [39,40]. However, the potential for compensatory mechanisms and the risk of systemic toxicity must be addressed. Future studies focusing on the development of more selective PDK1 inhibitors, along with combination therapies, will be necessary to optimize the therapeutic efficacy of PDK1 inhibition.Pan-cancer analysis found that PDK1 is abnormally overexpressed in various types of malignant tumors and is a poor prognostic factor for various types of malignant tumors. Experimental also found that enforced expression of PDK1 promoted osteosarcoma cell proliferation [41]. Currently, although there have been reports on the role of PDK1 in osteosarcoma, there is no study on the association between PDK1 and glycolic metabolism in osteosarcoma. Meanwhile, in this study, we first analyzed the clinical data of osteosarcoma and found that the expression level of PDK1 was negatively correlated with tumor progression and prognosis of osteosarcoma in both the total sample and non-metastatic group samples. GO and KEGG analysis showed that PDK1 and its highly positively correlated genes also promote tumor progression. Therefore, targeting PDK-1 is an attractive therapeutic target for osteosarcoma, and by inhibiting the function of PDK1, it can effectively inhibit the proliferation and metastasis of osteosarcoma cells. In the metastatic osteosarcoma group, however, we have not found the significant correlation between the expression level of PDK1 and osteosarcoma’s prognosis. This may be due to the small sample size of the metastatic group, which cannot reflect the true situation of the metastatic osteosarcoma and requires larger sample size validation. Given the variable metabolic dependencies and microenvironmental contexts of different cancers, PDK1’s prognostic significance likely hinges on the extent of glycolytic reliance, hypoxia, oncogenic drivers, and immune landscape—underscoring its potential as a context-dependent biomarker and therapeutic target in metabolically plastic tumors such as osteosarcoma. Although elevated PDK1 expression correlates with poor prognosis in osteosarcoma, this study does not establish PDK1 as a validated clinical biomarker. Future work involving large, well-characterized patient cohorts will be necessary to assess its utility in diagnosis, prognosis, or therapeutic response prediction.

Additionally, while our findings highlight the potential of PDK1 as a candidate target, they also raise questions regarding its role in the tumor microenvironment. Notably, PDK1 is known to influence immune cell function in some cancers. However, in our study, we chose not to delve into the immune-related data, as the primary focus was on its role in metabolic reprogramming.Future research could explore the interplay between PDK1 and immune cell infiltration in osteosarcoma, which may further illuminate its therapeutic potential in combination with immune checkpoint inhibitors.

## Conclusion

In this study, the relationship between PDK1 and the metabolism of osteosarcoma cells and the clinical prognosis of patients were explored through bioinformatics. PDK1 expression is closely associated with poor prognosis in osteosarcoma patients, especially those without metastasis. Its critical role in regulating glycolysis and cell proliferation underscores the potential of PDK1 as both a prognostic biomarker and a candidate target.Future studies focusing on selective inhibition of PDK1 and its role in immune modulation could open new avenues for the treatment of osteosarcoma and other cancers driven by metabolic reprogramming.

## Supporting information

S1 File Pan-cancer analysis PDK1 expression process. (PDF)

S2 File Western blotting original gel images. (PDF)

S1 Table The detailed clinical information of OS. (XLSX)

S2 Table Primer sequence information for PDK1 and GAPDH. (XLSX)

S3 Table The expression matrix of OS patients. (XLSX)

S4 Table Data processing record of PDK1 in OAS. (XLSX)

S5 Table PDK1 related genes. (XLSX)

S6 Table The data processing record of GSEA. (XLSX)

S7 Table The detailed analysis results of cancer grading. (XLSX)

S8 Table The analysis results of western-blotting. (XLSX)

S9 Table The processing results of PDK1 on glycolytic metabolism. (XLSX)

S10 Table The processing results of PDK1 on glycolytic metabolism. (XLSX)

## Abbreviation:

PDK1: 3-Phosphoinositide-Dependent Protein Kinase 1
FRT: Time to First Relapse in Days
PSP: Primary Site Progression
MS: Metastasis Status
FE: First Event
ROC: Receiver-Operating Characteristic Curve
AUC: Area Under the Curve
GO: Gene Ontology
KEGG: Kyoto Encyclopedia of Genes and Genomes
PDH: pyruvate dehydrogenase
GEPIA: Gene Expression Profiling Interactive Analysis
ACC: Adrenocortical carcinoma
BLCA: Bladder Urothelial Carcinoma
BRCA: Breast invasive carcinoma
CESC: Cervical squamous cell carcinoma and endocervical adenocarcinoma
COADREAD: Colon adenocarcinoma/Rectum adenocarcinoma Esophageal carcinoma
DLBC: Lymphoid Neoplasm Diffuse Large B-cell Lymphoma
ESCA: Esophageal carcinoma
GBM: Glioblastoma multiforme
GBMLGG: lower grade glioma and glioblastoma
HNSC: Head and Neck squamous cell carcinoma
KICH: Kidney Chromophobe
KIRC: Kidney Clear Cell Carcinoma
KIPAN: Pan-kidney cohort (KICH+KIRC+KIRP)
LGG: Brain Lower Grade Glioma
LIHC: Liver hepatocellular carcinoma
LUAD: Lung Adenocarcinoma
LUSC: Lung Squamous Cell Carcinoma
MESO: Mesothelioma
PAAD: Pancreatic adenocarcinoma
PCPG: Pheochromocytoma & Paraganglioma
SKCM: Skin Cutaneous Melanoma
THCA: Thyroid carcinoma
UCEC: Uterine Corpus Endometrial Carcinoma
UVM: Uveal Melanoma
