# Supplementary material for: PDK1, associated with glycolytic metabolism, is a potential prognostic biomarker in osteosarcoma
Source: PLoS One. 2025 Sep 19;20(9):e0332494. doi: 10.1371/journal.pone.0332494 (PMC12448963; doi:10.1371/journal.pone.0332494)
Supplement: S1 File — (PDF) [file pone.0332494.s001.pdf]

Dataset information:
We are from UCSC（ https://xenabrowser.net/ ）We downloaded the standardized pan cancer dataset TCGA TARGET GTEx (PANCAN, N=19131, G=60499) from the database, and further extracted the expression data of ENSG0000152256 (PDK1) gene in various samples. Furthermore, we screened samples from Solid Tissue Normal, Primary Solid Tumor, Primary Tumor, Normal Tissue, Primary Blood Derived Cancer Bone Marrow, Primary Blood Derived Cancer Peripheral Blood, and more. Further log2 (x+0.001) transformation was performed on each expression value, and finally, we excluded cancer species with less than 3 samples in a single cancer species. Finally, we obtained expression data for 34 cancer species, as shown in the Table.
Statistical drawing information:
We used R software (version 3.6.4) to calculate the expression differences between normal and tumor samples in each tumor, and conducted significance analysis using non paired Wilcoxon Rank Sum and Signed Rank Tests. We observed significant upregulation in 21 types of tumors, such as
GBM(Tumor:2.75±0.91,Normal:0.89±1.25,p=3.3e-63)、
GBMLGG(Tumor:1.66±1.02,Normal:0.89±1.25,p=5.1e-40)、
LGG(Tumor:1.33±0.80,Normal:0.89±1.25,p=2.6e-14)、
UCEC(Tumor:2.50±1.09,Normal:1.36±0.42,p=2.4e-8)、
LUAD(Tumor:2.85±0.86,Normal:1.90±0.88,p=1.3e-56)、
ESCA(Tumor:3.56±0.82,Normal:2.12±1.17,p=1.3e-64)、
STES(Tumor:3.10±0.86,Normal:2.04±1.26,p=2.1e-89)、
KIPAN(Tumor:3.19±1.43,Normal:1.98±1.31,p=9.5e-24)、
COAD(Tumor:2.61±0.72,Normal:1.96±1.51,p=2.7e-15)、
COADREAD(Tumor:2.58±0.71,Normal:1.99±1.50,p=4.9e-15)、
STAD(Tumor:2.90±0.80,Normal:1.80±1.48,p=5.0e-33)、
HNSC(Tumor:3.02±0.94,Normal:1.90±0.70,p=3.4e-13)、
KIRC(Tumor:4.03±0.95,Normal:1.98±1.31,p=2.6e-63)、
LUSC(Tumor:3.84±0.82,Normal:1.90±0.88,p=1.2e-124)、
LIHC(Tumor:1.59±1.09,Normal:1.42±1.05,p=0.01)、
WT(Tumor:3.42±0.75,Normal:1.98±1.31,p=3.0e-29)、
BLCA(Tumor:2.32±0.90,Normal:2.01±0.56,p=0.02)、
PAAD(Tumor:1.96±0.80,Normal:0.56±1.35,p=7.9e-36)、
UCS(Tumor:3.07±1.00,Normal:2.11±0.62,p=8.1e-10)、
ALL(Tumor:2.94±0.94,Normal:1.26±1.30,p=4.9e-32)、
LAML(Tumor:3.90±0.86,Normal:1.26±1.30,p=7.2e-63),
We found significant downregulation in 7 types of tumors, such as
BRCA(Tumor:2.45±0.95,Normal:2.59±0.63,p=2.3e-3)、
SKCM(Tumor:1.72±1.43,Normal:3.81±0.75,p=5.2e-42)、
THCA(Tumor:1.31±0.64,Normal:1.78±0.94,p=1.2e-26)、
TGCT(Tumor:2.95±0.66,Normal:3.88±0.82,p=3.3e-23)、
PCPG(Tumor:2.03±0.90,Normal:3.59±0.84,p=0.01)、
ACC(Tumor:2.07±1.55,Normal:2.51±1.38,p=0.01)、
KICH(Tumor:1.43±0.94,Normal:1.98±1.31,p=9.0e-6).
