# Supplementary figures and images for: PDK1, associated with glycolytic metabolism, is a potential prognostic biomarker in osteosarcoma
Source: PLoS One. 2025 Sep 19;20(9):e0332494. doi: 10.1371/journal.pone.0332494 (PMC12448963; doi:10.1371/journal.pone.0332494)

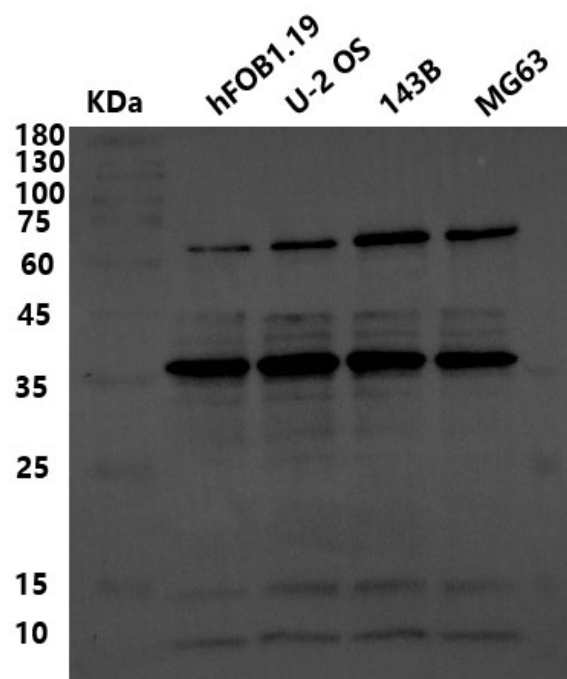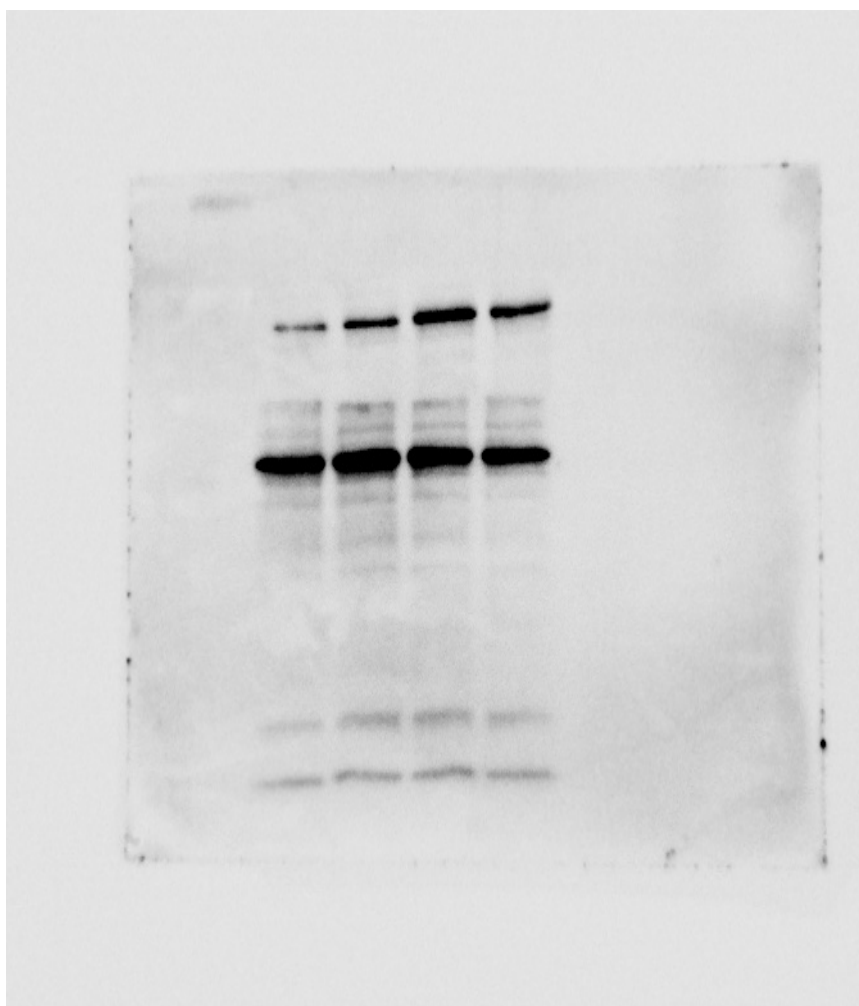

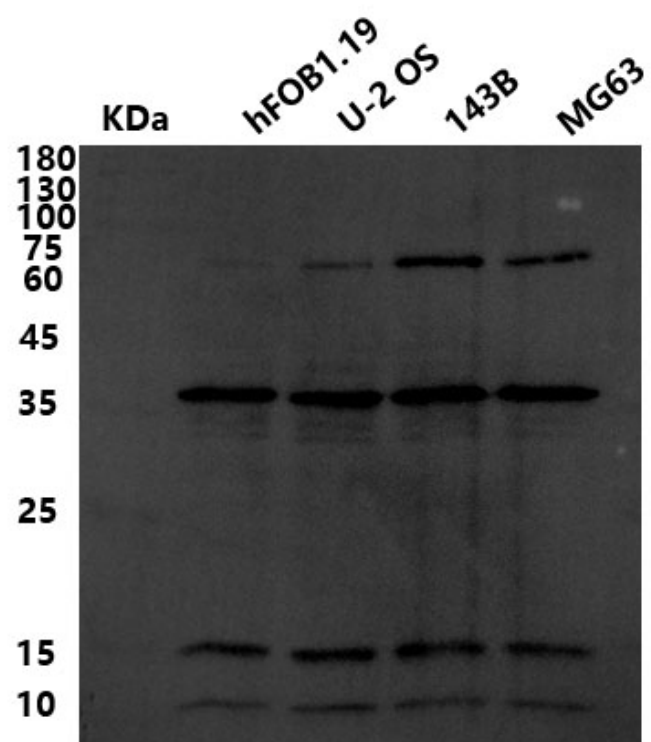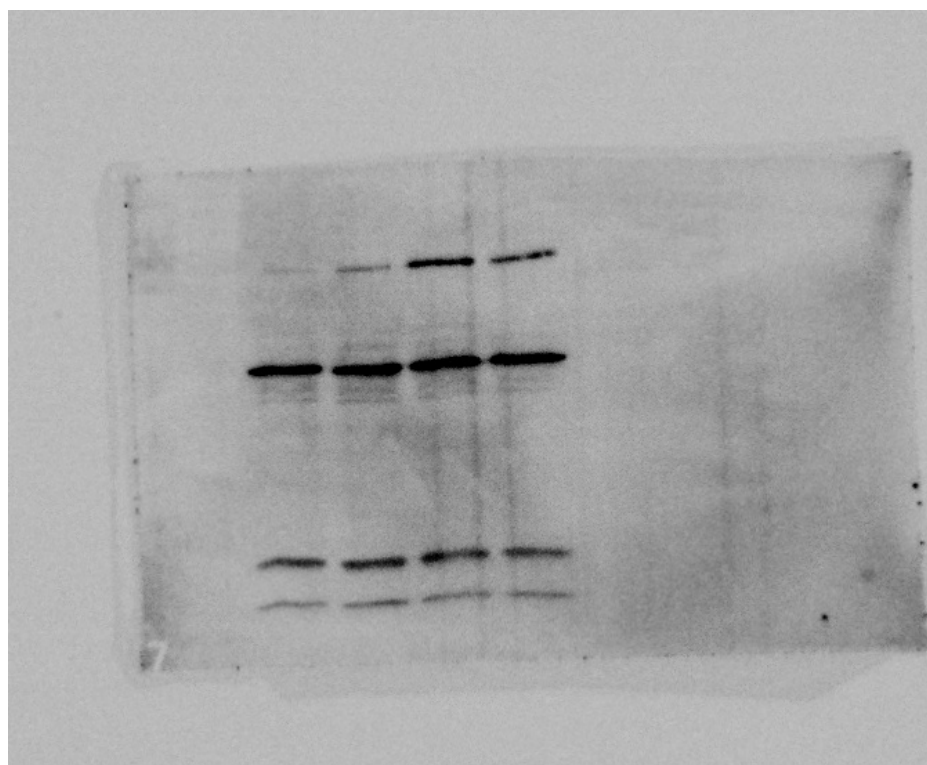

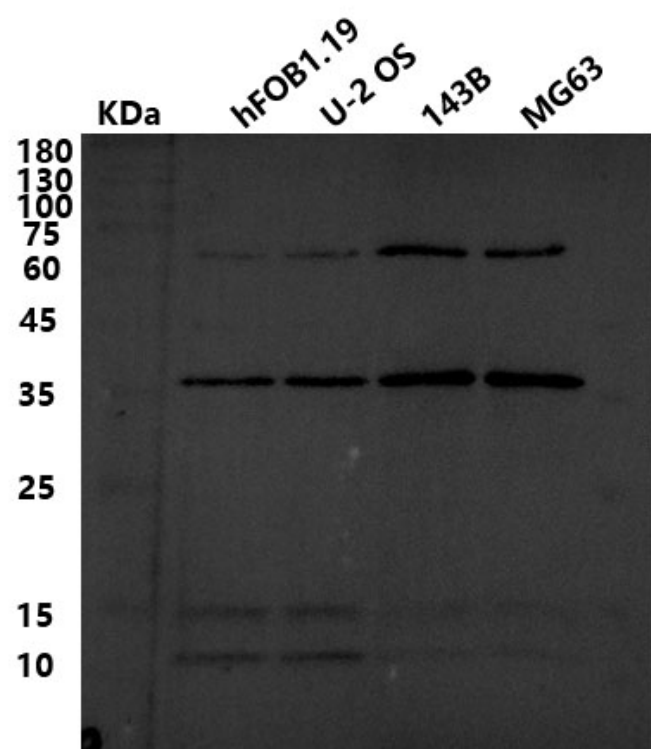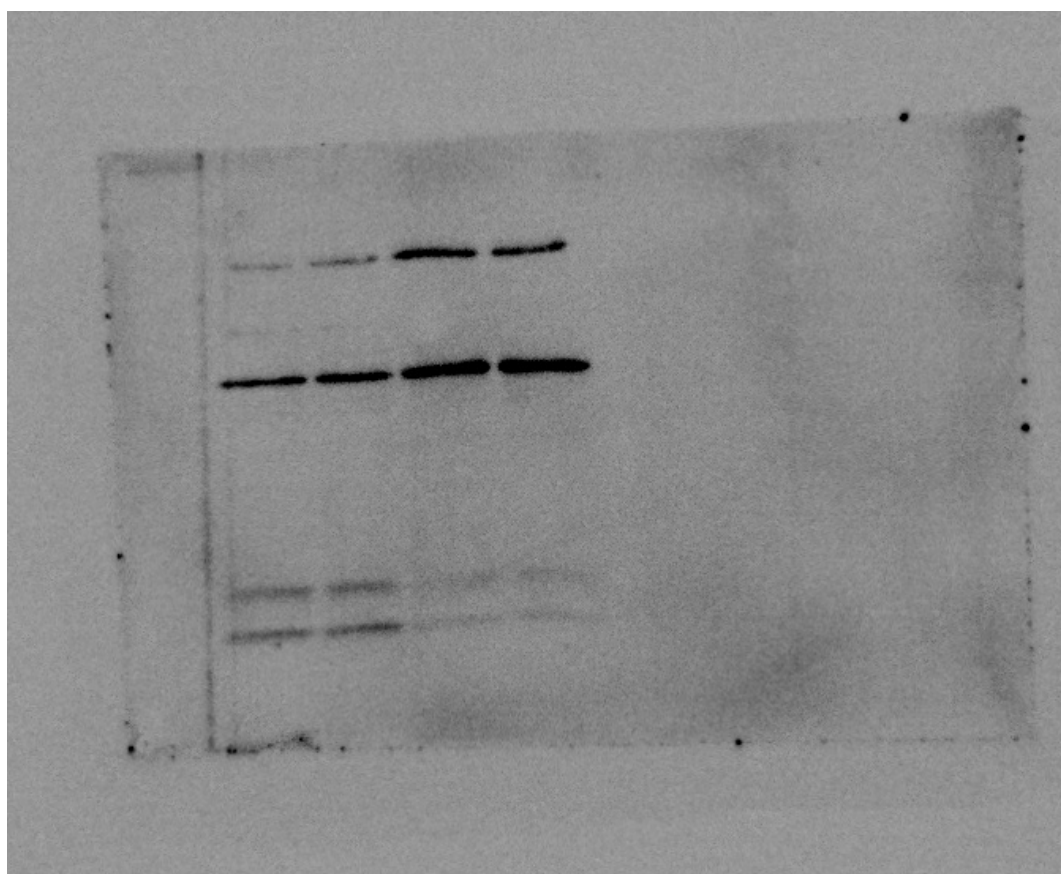

Supplement: S2 File — (PDF) [file pone.0332494.s002.pdf]
